# Patient-Specific Simulation of Pneumoperitoneum for Laparoscopic Surgical Planning

**DOI:** 10.1007/s10916-019-1441-z

**Published:** 2019-09-10

**Authors:** Shivali Dawda, Mafalda Camara, Philip Pratt, Justin Vale, Ara Darzi, Erik Mayer

**Affiliations:** Department of Surgery and Cancer, Imperial College London, London, UK

**Keywords:** Simulation, Pneumoperitoneum, Patient-specific, Laparoscopy, Surgical planning

## Abstract

Gas insufflation in laparoscopy deforms the abdomen and stretches the overlying skin. This limits the use of surgical image-guidance technologies and challenges the appropriate placement of trocars, which influences the operative ease and potential quality of laparoscopic surgery. This work describes the development of a platform that simulates pneumoperitoneum in a patient-specific manner, using preoperative CT scans as input data. This aims to provide a more realistic representation of the intraoperative scenario and guide trocar positioning to optimize the ergonomics of laparoscopic instrumentation. The simulation was developed by generating 3D reconstructions of insufflated and deflated porcine CT scans and simulating an artificial pneumoperitoneum on the deflated model. Simulation parameters were optimized by minimizing the discrepancy between the simulated pneumoperitoneum and the ground truth model extracted from insufflated porcine scans. Insufflation modeling in humans was investigated by correlating the simulation’s output to real post-insufflation measurements obtained from patients in theatre. The simulation returned an average error of 7.26 mm and 10.5 mm in the most and least accurate datasets respectively. In context of the initial discrepancy without simulation (23.8 mm and 19.6 mm), the methods proposed here provide a significantly improved picture of the intraoperative scenario. The framework was also demonstrated capable of simulating pneumoperitoneum in humans. This study proposes a method for realistically simulating pneumoperitoneum to achieve optimal ergonomics during laparoscopy. Although further studies to validate the simulation in humans are needed, there is the opportunity to provide a more realistic, interactive simulation platform for future image-guided minimally invasive surgery.

## Introduction

Image guidance systems in surgery offer great potential to increase surgical accuracy and safety by augmenting the visualization of anatomical landmarks and subsurface structures during minimally invasive procedures. The utility of such technologies is often limited in laparoscopy due to the creation of pneumoperitoneum, which shifts the skin and deforms the abdominal wall, organs and blood vessels [1–3]. This also makes it challenging to ensure the optimal positioning of trocars, which is an essential determinant of the operative quality, safety and ease of laparoscopy that is presently based on the surgeon’s experience and judgment of the post-insufflation operative field. Improper placement can result in poor laparoscopic view or instrumentation and poses an increased risk of vascular or organ damage. Modeling the changes that occur in the abdomen with gas insufflation is one way to overcome these issues, as well as facilitate surgical planning by providing a realistic, three-dimensional representation of the intraoperative scenario. It also offers the opportunity to enhance surgical training simulators and allow for guidance of trocar positioning in a way that optimizes the ergonomics of laparoscopic instrumentation.

Only a handful of groups have proposed methods for simulating pneumoperitoneum, from which a sufficient or desired technology has yet to surface. Previous groups have used traditional, physically-based methods of modeling dynamic objects which use internal and external forces to determine the positions of the displaced objects by time-integrating accelerations [4–8]. These techniques are typically highly complex and involve long computational times. In this work, biomechanical deformation is modeled with a position-based dynamics (PBD) approach, which simulates dynamic systems by calculating the displacement of objects to valid new positions such that constraints are satisfied [9]. As PBD works directly on the positions of objects (rather than with forces), it offers unconditional stability and can compute manipulations at interactive speeds (i.e. in real-time) with high visual fidelity that is especially suitable for complex surgical simulations [9]. Furthermore, using PBD entails a faster, more efficient data preparation protocol that favors simulation on a patient-to-patient basis, whereas other approaches are highly time consuming and therefore not as feasible nor efficient for patient-specific planning. As the profile and extent of deformation to the abdominal wall and organs vary depending on each individual’s physique, patient-specific modeling is highly advantageous.

This work is aimed at developing a platform that simulates the anatomical changes resulting from gas insufflation during laparoscopy in a patient-specific manner, using preoperative CT scans as the input data. This can assist surgeons in the planning and rehearsal of laparoscopic procedures by allowing realistic visualization and interaction with a virtual, 3-dimensional (3D) model of a specific patient’s anatomy, post-insufflation. It will further serve to guide trocar positioning in a way that optimizes the ergonomics of laparoscopic instrumentation. This would allow for greater accuracy and utility of preoperative planning, which should ultimately improve surgical performance, decrease operation times and reduce error [10]. The simulation will be developed using a PBD approach on a porcine model, for practicability of obtaining insufflated and deflated volumetric data. Its feasibility for modeling insufflation in humans will be subsequently assessed by correlating the virtual simulated pneumoperitoneum to real post-insufflation measurements obtained from patients in theatre.

## Materials and methods

3D models were generated from two sets of porcine data: insufflated scans and deflated scans. Models derived from the insufflated CT scans were considered ground truth. An artificial pneumoperitoneum was simulated on models from the deflated scans. The simulation parameters were optimized by comparing its output against the real pneumoperitoneum (derived from the insufflated porcine scans), and minimizing the difference.

### Data preparation: 3D reconstruction and mesh generation

The datasets used were originally collected for other purposes in accordance with institutional guidelines, under the appropriate licenses, permissions and ethical approval. Eight pigs underwent gas insufflation of up to 12 mmHg of abdominal pressure. Acquisition of contrast-enhanced CT images (2.5 mm slice thickness, 512 × 512 acquisition matrix) was carried out with the animals in supine position, and repeated after deflation to produce two datasets for each pig: a deflated and insufflated volume.

**Fig. 1:**
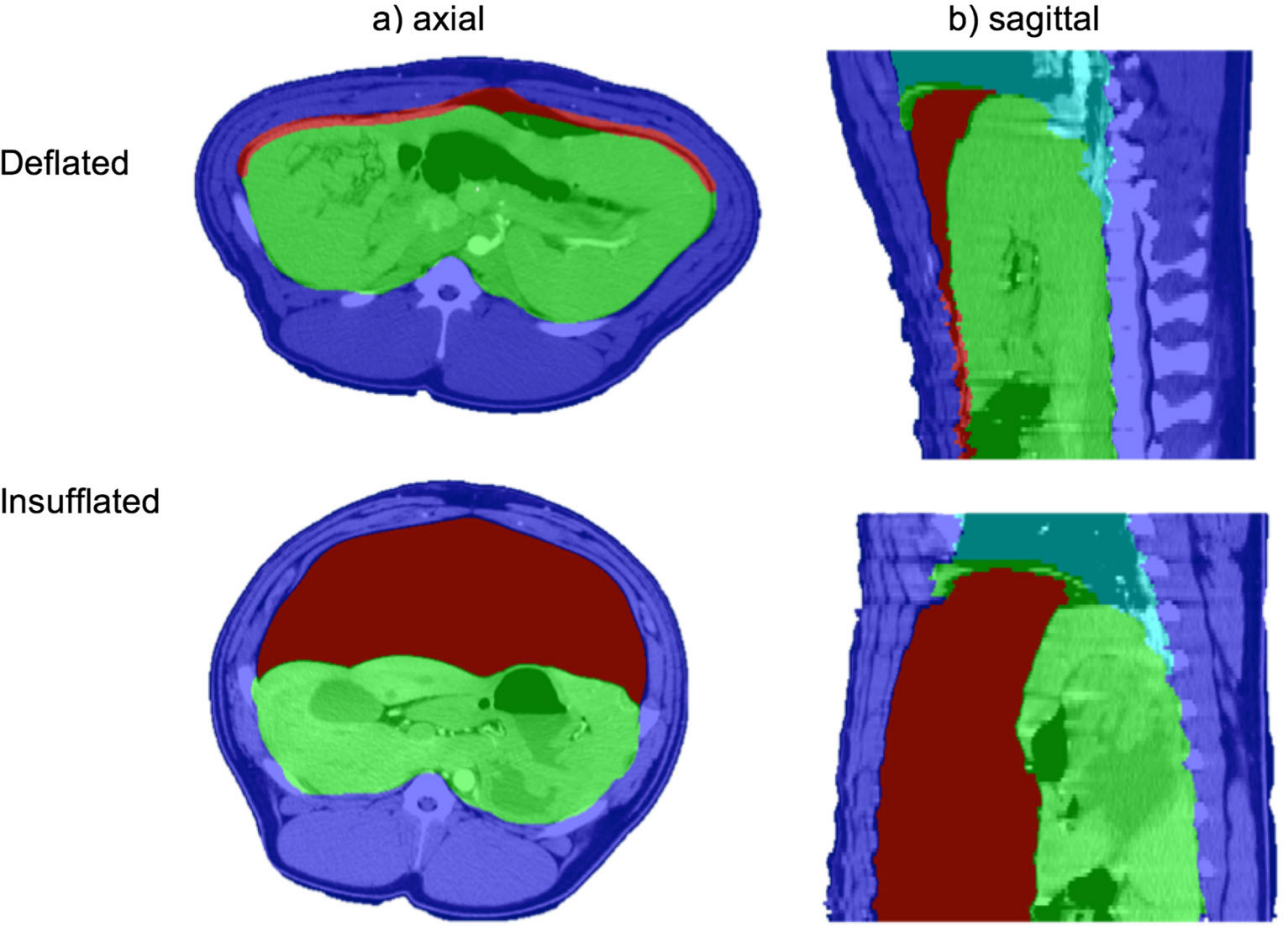
Slices from insufflated and deflated porcine CT scans demonstrating the segmentation of different regions: gas (red), abdominal viscera (green), lungs (light blue) and abdominal wall (dark blue). The red line in the deflated scans indicates the boundary between the peritoneal cavity and the abdominal wall

**Fig. 2:**
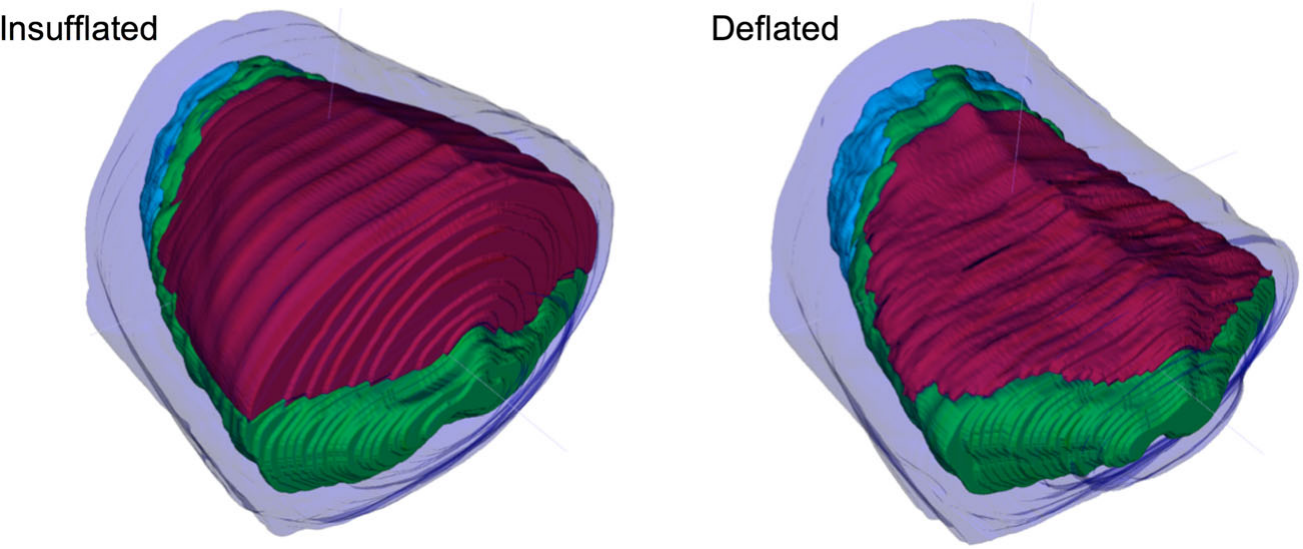
3D reconstructions of an insufflated and deflated porcine scan, produced by interpolating individually segmented axial slices in Fig. 1a

3D reconstruction of preoperative scans can be produced through the process of manual or semi-automatic ‘segmentation’, whereby particular regions on a series of medical images are highlighted in different colors and interpolated in three dimensions to create a virtual model of a specific patient’s anatomy (Figs. 1 and 2). Computed tomography (CT) provides sufficient information for abdominal reconstruction as the high spatial resolution prevents underlying tissues and structures from being superimposed [11]. 3D volume data was generated by segmenting axial slices of the original porcine CT scans in ITK-SNAP v3.6.0 [12] and extrapolating the model into a closed structure. CT images were divided into four regions (Fig. 1): the abdominal-thoracic wall (dark blue), abdominal viscera (green), pneumoperitoneum (red) and lungs (light blue). The rationale for segmenting organs collectively was based on previous attempts at simulating pneumoperitoneum, which produced acceptable results from modeling the abdominal viscera as a single homogenous structure [4–6].

**Fig. 3:**
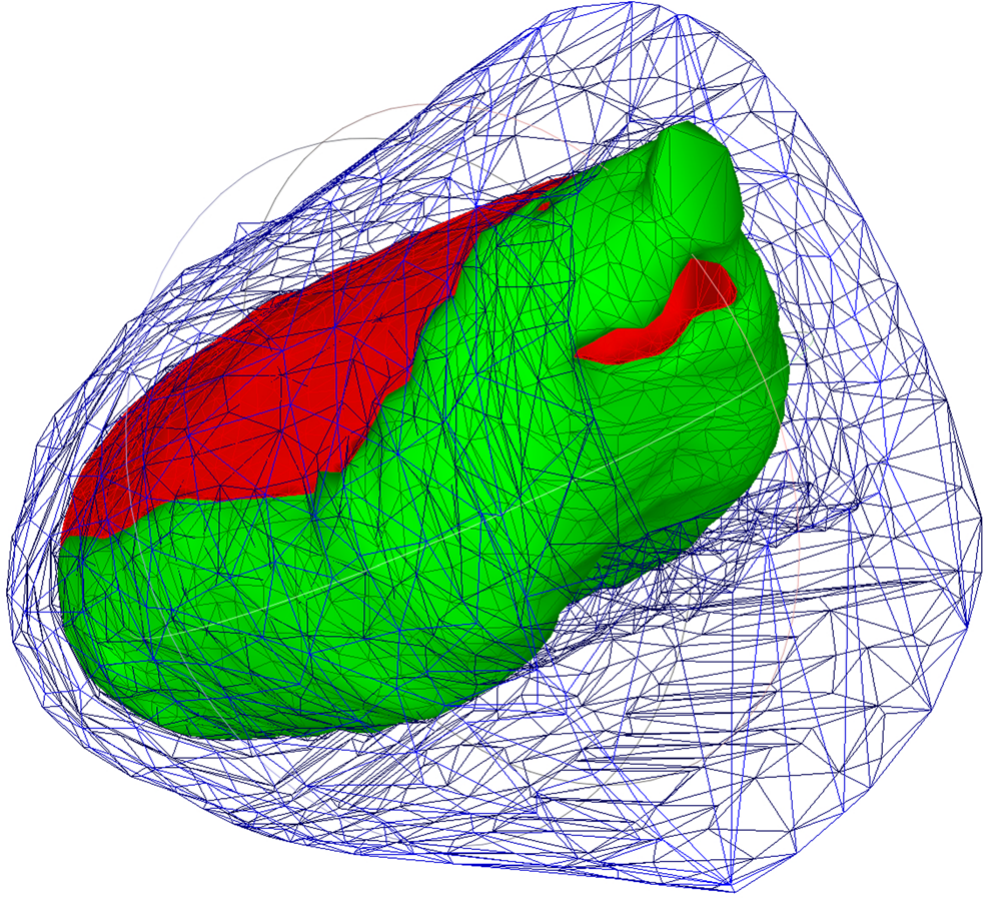
Triangular surface meshes of insufflated porcine (abdominal wall in blue, organs in green, pneumoperitoneum in red)

3D segmentations were exported from ITK-SNAP into MeshLab (v2016.12) as triangular STL surface meshes (Fig. 3), where they were simplified (to around 10,000 triangular faces) and scaled down to half their size in order to speed up the calibration by inputting fewer particles for simulation, whilst preserving particle size [13].

### Simulation

**Fig. 4:**
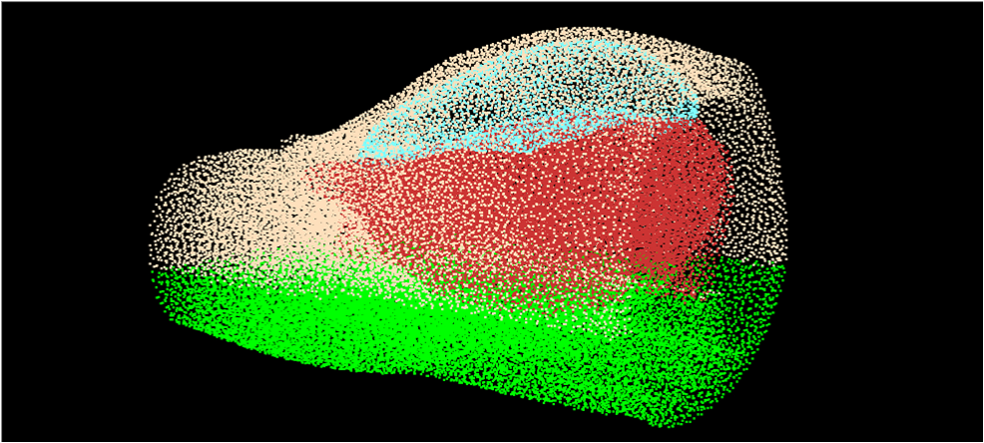
Particle density of insufflated mesh, separated into the inflatable structure (blue), skin (peach), viscera (red). Green points indicate fixed regions in the back

**Fig. 5:**
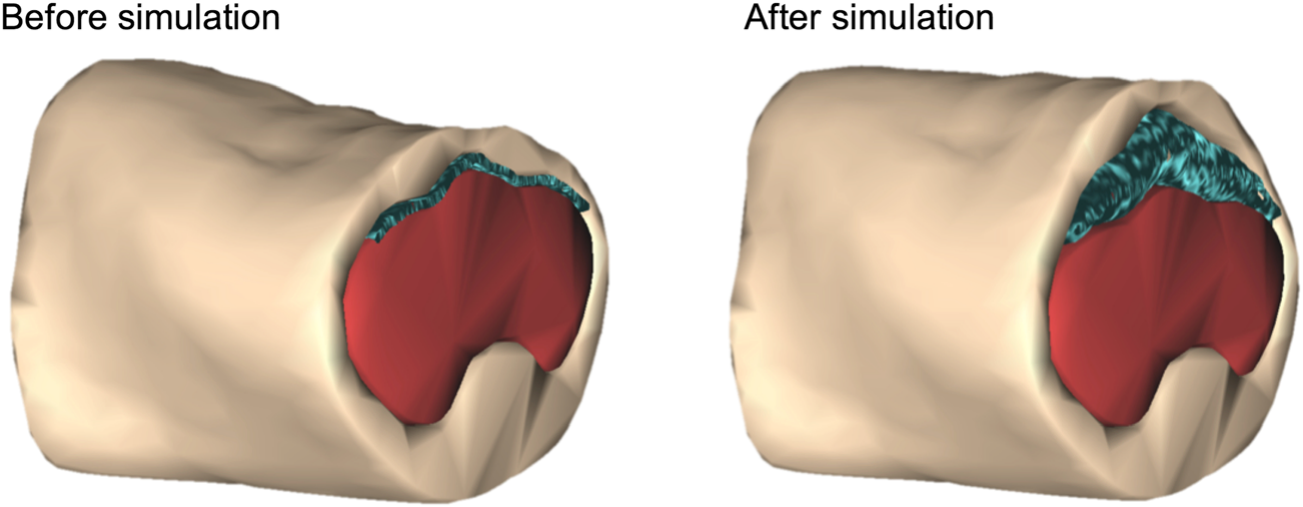
Pneumoperitoneum before and after simulation, showing increased volume of the pneumoperitoneum (10x) and resultant organ compression and abdominal wall deformation

The abdominal wall and viscera were considered soft bodies, and the boundary between them regarded as an ‘inflatable structure’. Each of these entities were entirely discretised into particles (Fig. 4) and modeled as separate structures by applying different simulation parameters to the particles. The inflatable structure was derived from highlighting the boundary between the abdominal wall and viscera in the segmentation step. This region represents the peritoneal cavity where gas is insufflated in laparoscopy, and is artificially inflated in the simulation by applying pressure from within the mesh (Fig. 5).

Gravity in the simulation was set to zero, and particles in a specific region of the back (3 mm below the axis defined by the center of mass) were fixed along the cranio-caudal axis in order to account for contact with the operating table (green points in Fig. 4). An exhaustive search was conducted to determine the optimal combination of parameters that would minimize the error in the simulated inflatable structure, when compared to the ground truth model derived from the insufflated porcine scan. Ranges adopted for each parameter were selected based on previous work involving soft tissue calibration [14] and experience of the simulation’s sensitivity to certain parameters. Table 1 shows the simulation settings used during this calibration process.

**Table 1:** Simulation settings for calibration of parameters

Time step	1/60s
Simulation substeps	3 (collision detections per timestep)
Substep iterations	9
Cluster spacing factor	3.33 (controls cluster overlapping)
Volume sampling factor	4 (controls particle density)
Relaxation type	Local (relaxation factor = 1.0)
Acceleration due to gravity	0 m/s^2^
Tissue density	1.05 g/cm^3^
Shape friction coefficient	0.35
Particle friction coefficient	0.25
Damping factor	12.0

### Optimization using porcine data

Table 2 lists the parameters undergoing optimization. The pressure applied on the inflatable is increased and the simulation is performed for each value of pressure (from 1.0 to 10.0), which represents a proportionate increase in the original volume of the inflatable. This can in future be translated to a value of pressure in mmHg via a second calibration process whereby scans acquired over a range of insufflation pressures would provide a ground truth model for validation at each pressure.

**Table 2:** Parameters for optimization

Parameter	Influence	Range
Cluster stiffness	Controls stiffness and deformability of soft tissues	0.4, 0.5, 0.6, 0.7, 0.8
Spring stiffness	Controls stiffness of the inflatable structure and its resultant deformability	0.1, 0.2, 0.3, 0.4, 0.5, 0.6, 0.7, 0.8, 0.9, 1.0
Particle radius	Determines the size of each particle, directly influencing the number of particles that comprise an object	2.2 mm, 2.7 mm, 3.3 mm
Simulation pressure	Proportionate to the increase in the original volume of the inflatable	1.0, 1.5, 2.0, 2.5, 3.0, 3.5, 4.0, 4.5, 5.0, 5.5, 6.0, 6.5, 7.0, 7.5, 8.0, 8.5, 9.0, 9.5, 10.0, 10.5, 11.0, 11.5, 12.0, 12.5, 13.0, 13.5, 14.0, 14.5, 15.0

The simulation was optimized by adopting the set of parameters that returned the minimal error when comparing simulated meshes with those derived from the insufflated porcine scans (ground truth). This comparison was made by calculating the mean Euclidean distances between corresponding points on the simulated meshes and the ground truth meshes across all vertices, for the entire porcine dataset. It is this quantity that underwent minimization during optimization. The resultant set of parameters are summarized in Table 3.

**Table 3:** Optimized parameters

Parameter	Optimal value
Cluster stiffness	0.6
Spring stiffness	0.5
Particle radius	2.7 mm
Simulation pressure	8.9

To avoid undesirable behavior, such as the pneumoperitoneum expanding outside the abdominal wall, extra springs were added into the simulation to assure connectivity between the inflatable structure and the abdominal wall without invalidating the resulting set of optimized parameters. The optimized simulation was performed on each animal for the given value of pressure with which they were inflated during CT acquisition. Meshes representing the abdominal wall, the viscera and the pneumoperitoneum were extracted, as well as the mean error, standard deviation and minimum and maximum errors (Euclidian distances).

### Feasibility of modeling pneumoperitoneum in humans

**Fig. 6:**
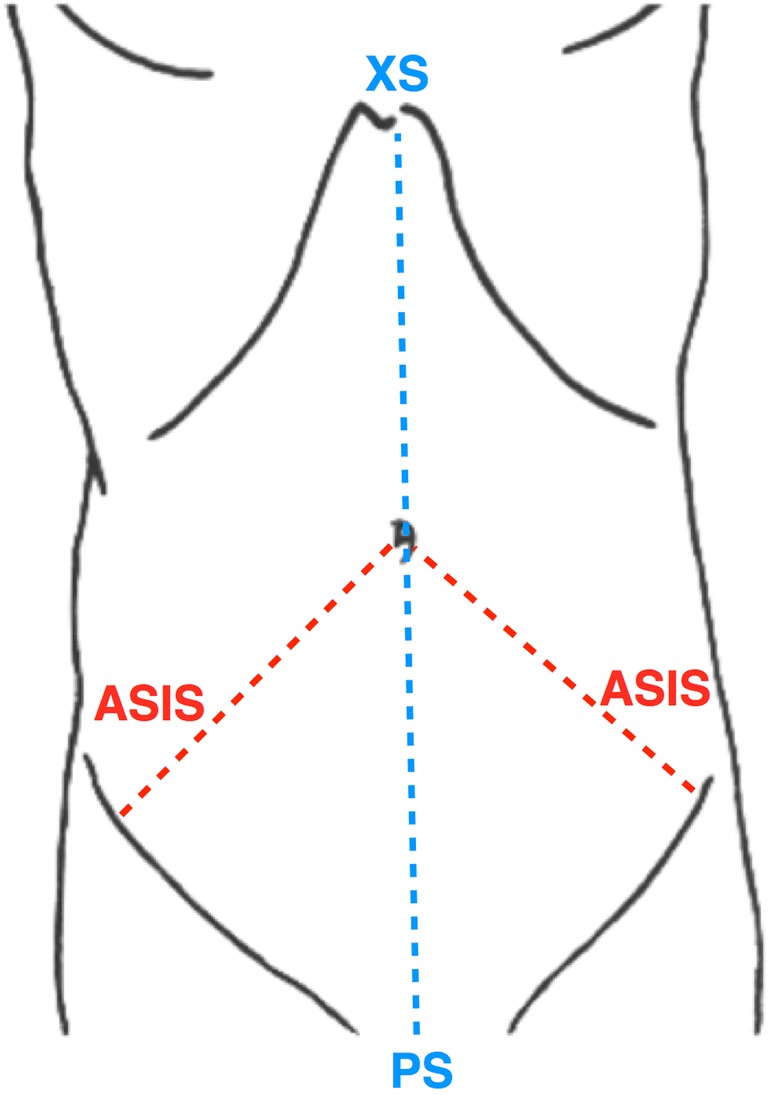
Three measurements were taken from landmarks on the abdominal surface: umbilicus to right and left anterior-superior iliac spines (ASIS), xiphisternum (XS) to pubic symphysis (PS)

As well as giving unnecessary exposure to radiation, it is impractical to scan patients whilst maintaining pneumoperitoneum for direct comparison to a simulation. Insufflation modeling in humans was therefore assessed by correlating the simulation’s output to real post-insufflation measurements obtained from patients in undergoing laparoscopic surgery (Fig. 6). Landmarks were chosen for their accessibility through sterile drapes and visibility on CT images. Under the existing ethical protocol ‘*Improving Outcomes in Robotic and Endoscopic Surgery using Augmented Reality Guidance*’ (REC reference 07/Q0703/24), informed and written consent was obtained from patients recruited to the study.

## Results

### Validation on porcine data

**Fig. 7:**
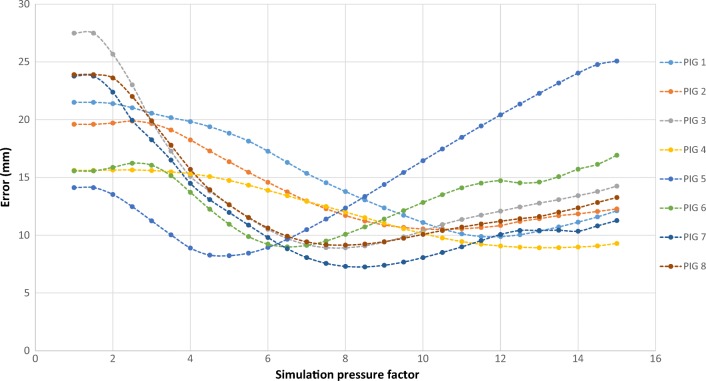
Mean overall error of simulated meshes across simulation pressure, as average distance to corresponding vertices on ground truth meshes from insufflated porcine scans

Using the resultant optimal set of parameters, pneumoperitoneum was simulated for each pig by increasing the volume of the inflatable structure. Volume is proportional to the simulation pressure and hence labeled “simulation pressure factor”. The overall mean error in the simulated meshes was determined by calculating the Euclidean distance between corresponding points on the simulated pneumoperitoneum and ground truth models. This was plotted for each pressure value, for each pig (Fig. 7). The simulation produced the best results in the 7th porcine dataset, which gave the lowest overall error (7.26 mm). Conversely, the 2nd dataset was the least successful, returning the highest overall error (10.5 mm). The initial displacement, calculated before any simulation pressure was applied, was 23.8 mm and 19.6 mm for the most and least accurate simulations respectively.

All datasets followed a general trend whereby the mean overall error decreased until it reached a minimum, at which point the simulated pneumoperitoneum was most aligned with the ground truth meshes. Increasing the pressure beyond this minimum began to increase the overall error, showing that the simulation was over-expanding the inflatable structure. Curves displayed variable behavior in reaching their minimum error at different simulation pressure values. Table 4 gives a summary of the most and least accurate simulations.

**Table 4:** Average error of most and least accurate simulations

Simulation	Most accurate (pig 7)	Least accurate (pig 2)
Average overall error (mm)	7.26	10.5
Standard deviation (mm)	2.15	2.77
Minimum (mm)	0.158	0.190
Maximum (mm)	15.2	16.2
Initial displacement – before simulation (mm)	23.8	19.6

**Fig. 8:**
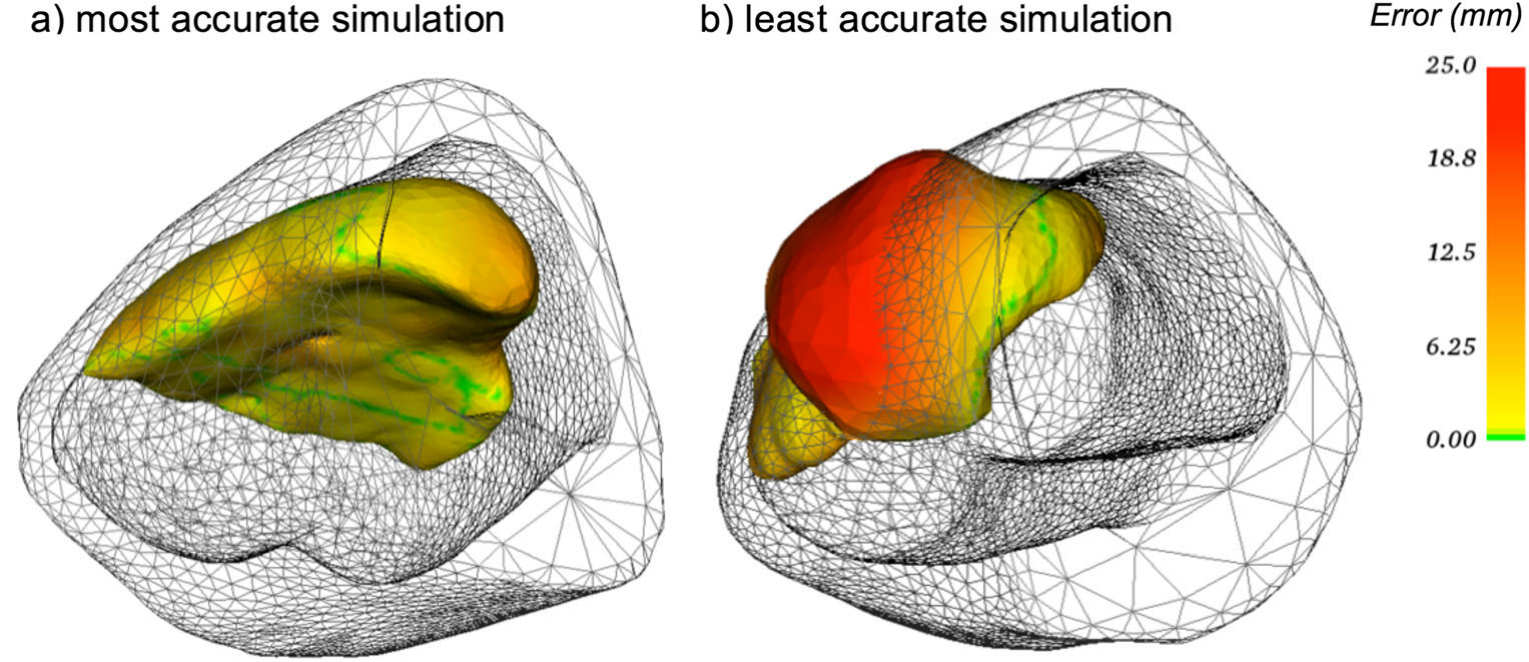
Color maps displaying map illustrating the overall error (mm) of the simulated pneumoperitoneum of the 2nd and 7th porcine datasets at their optimal pressure parameters. Colors correspond to the overall error in millimeters (as on the color bar). Warmer colors indicate a higher degree of misalignment with the ground truth mesh, implying greater overall error

Errors were derived using an absolute distance function and are illustrated in Fig. 8 on color maps of the simulated inflatable structures. The most and least successful simulations are shown for contrast; Fig. 8a illustrates the average error in the well-simulated 7th dataset (7.26 mm) whereas Fig. 8b demonstrates the same concept in the least accurate 2nd dataset (10.5 mm). Error in the 2nd dataset is evident in the red region, where the inflatable structure has expanded outside of the wireframe of the abdominal wall.

### Human simulation

Human CT scans were successfully segmented and simulated for pneumoperitoneum. Pre- and post-insufflation measurements were collected from theatre and from the generated anatomic models (Table 5).

**Table 5:** Abdominal surface changes from insufflation: human data

	Intraoperative data (cm)			Simulation (cm)		
	Umbilicus to ASIS-R	Umbilicus to ASIS-L	Xiphisternum to pubic symphisis	Umbilicus to ASIS-R	Umbilicus to ASIS-L	Xiphisternum to pubic symphisis
Normal	18	17.5	32.8	17.5	18.4	39.3
Insufflated	19	17.5	37.4	18.9	20.1	41.9
Change	1	0	4.6	1.4	2.3	2.6

## Discussion

Segmentations of the porcine dataset were sufficient to derive an optimal set of parameters for the simulation. The simulation was successful in realistically modeling organ and abdominal wall deformation, with an average error of 7.26 mm in the most accurate simulation. This “error” refers to the overall difference between the simulation’s output, when compared against the real-life inflated porcine. This must be interpreted in context of the original discrepancy between the insufflated and deflated porcine, which was calculated to be 23.8 mm. This initial displacement, present before any pressure was simulated, represents the “error” that surgeons currently need to operate with. The threefold reduction in error shows that the methods proposed here have provided a significantly improved picture of the intraoperative scenario.

The porcine model has good translatability for human simulation. Pigs are the preferred animate trainers for complex laparoscopic techniques as the size of their abdominal cavity and their foregut anatomy is similar to that of humans, which provides comparable ergonomics to human laparoscopy and allows for the creation of pneumoperitoneum [15]. The muscle layers that formed the boundaries of the abdominal cavity in this simulation are organized in a similar fashion in both pigs and humans [16].

A major issue in the field of patient-specific biomechanical modeling is how to reproduce clinically accurate simulations without knowledge of the patient-specific mechanical properties of tissues. Abdominal deformation by pneumoperitoneum varies by age, sex, BMI and other patient variables. However, Miller et al. demonstrate that it is possible to achieve, for the purpose of this application, a realistic prediction of tissue deformations using preoperative images alone [17]. A patient-specific anatomic response to increasing abdominal pressure can therefore be calculated using solely the geometry of the abdominal wall - which is obtained from the segmentation of preoperative CT image data as described. This suggests the effect of patient mechanics on abdominal deformation by pneumoperitoneum can be disregarded.

When compared to previous works, this simulation models pneumoperitoneum with respectable accuracy. Oktay et al. achieved an initial average error of 10.9 mm (before image registration) when validated on 3 porcine CT-scans [7]. Bano et al. simulated movement of the abdominal wall and viscera with 5 mm and 6 mm accuracy respectively from validation in 2 pigs [5], and Nimura et al. report an average error of 26.9 mm from comparing their models to the displacement of optically-sensed points on human abdominal surface [8]. The minimum and maximum average errors obtained from this simulation was 7.26 mm and 10.5 mm respectively. These results were obtained from a much larger dataset (eight pigs) than any previous work. It provides the added speed and unconditional stability of PBD, which gives the simulation promising applications due to its high visual fidelity and ability to compute deformations in real-time. Furthermore, a realistic, patient-specific simulation of human pneumoperitoneum has been demonstrated using a technology that works at interactive speeds that is feasible for large-scale use.

This study has some weaknesses. Certain sources of error may have contributed to simulation inaccuracy: the study protocol involved a large amount of data processing, which is liable to the loss of precision despite being handled mostly by the same individual. To preserve the simulation’s generalizability, pigs were not normalized for size during calibration, which may reflect in the data: results suggest a possible relationship between elements of the pig’s geometrical features and the optimal simulation parameters. Furthermore, a robust validation for human simulation is required. This could be achieved by acquiring more accurate measurements of pneumoperitoneum, such as intraoperative imaging or optical position sensing of markers placed on the abdominal surface [3, 8]. Regardless, this work has demonstrated that human preoperative medical images can be successfully processed for real time, 3D, patient-specific simulation.

The simulation’s performance can be improved through various approaches. Future studies could acquire CT scans across a range of different insufflation pressures to ensure there is accurate modeling of the rate of organ deformation. Furthermore, as the simulation was developed *and* tested on the same dataset of eight pigs, it should be validated on a new dataset. Incorporating a gravity-compensation study would enable the framework to simulate pneumoperitoneum even when the patient is lying on their side, despite CT acquisition of them lying supine. This would increase its application to a variety of positions – e.g. in urology, where patients are positioned on their side for laparoscopic nephrectomies. Also, modeling the organs individually could produce greater accuracy that would be beneficial for more detailed surgical image guidance (e.g. for liver resections).

Next steps would involve using the simulation to inform and enhance surgical planning. The simulation could be integrated into virtual reality simulators used in the training of laparoscopic surgeons to create a highly realistic, patient-specific training environment for operation rehearsal [18]. The stability and speed of PDB allows surgeons to interact with realistically insufflated, virtual models of patient anatomy in real time, giving them the opportunity to define and rehearse their surgical strategy on a case-by-case basis depending on the patient and target organ. Augmented reality (AR), which involves the addition of virtual elements to a real scene, has recently become a popular area of development in the laparoscopic community and in image-guided surgery. However, the utility of AR in laparoscopy is largely limited: insufflating the abdomen complicates image registration and makes intraoperative anatomy inconsistent to 3D reconstructions of preoperative scans [19]. Modeling insufflation offers the opportunity to overcome these discrepancies, which have been a major obstacle in the use of AR as an image guidance tool in laparoscopy so far [20].

Altogether, this highlights how image guidance systems in laparoscopy could hugely benefit from patient-specific simulation of pneumoperitoneum. This research presents a method for realistically simulating pneumoperitoneum using preoperative images as the input data. It aims to facilitate surgical planning, as well as provide a more realistic platform for future image guidance in laparoscopy.
